# Complementary strategies in pancreatic cancer precision medicine: therapeutic prediction and immune modulation

**DOI:** 10.3389/fonc.2025.1718911

**Published:** 2025-12-03

**Authors:** Wonmin Lee, Hyun-Myung Woo, Ja Hyang Cho, Man S. Kim

**Affiliations:** 1 Translational-Transdisciplinary Research Center, Medical Science Research Institute, Kyung Hee University Hospital at Gangdong, College of Medicine, Kyung Hee University, Seoul, Republic of Korea; 2 Department of Medicine, Kyung Hee University College of Medicine, Seoul, Republic of Korea; 3 Department of Biomedical & Robotics Engineering, College of Engineering, Incheon National University, Incheon, Republic of Korea; 4 Department of Pediatrics, Kyung Hee University Hospital at Gangdong, College of Medicine, Kyung Hee University, Seoul, Republic of Korea

**Keywords:** pancreatic ductal adenocarcinoma, patient-derived organoids, mRNA neoantigen vaccines, precision medicine, KRAS mutation, immunotherapy, drug resistance

## Abstract

**Background:**

Pancreatic ductal adenocarcinoma (PDAC) remains highly lethal with five-year survival below 10%, primarily due to late diagnosis, treatment resistance, and immunosuppressive microenvironment.

**Methods:**

This review examines recent advances in PDAC precision medicine by analyzing organoid-based drug screening platforms, mRNA immunotherapy developments, and integrated diagnostic technologies.

**Results:**

Patient-derived organoids demonstrate strong predictive value for clinical outcomes, with drug sensitivity profiles correlating with patient responses (concordance >80%). Personalized mRNA neoantigen vaccines induce robust T-cell responses, with vaccine responders showing significantly prolonged recurrence-free survival (median not reached vs. 13.4 months). KRAS-targeted therapies achieve 10-15% response rates in G12C-mutant PDAC. Integration of spatial transcriptomics and liquid biopsy enables real-time molecular monitoring.

**Conclusions:**

The development of patient-derived organoids for drug sensitivity testing, personalized mRNA vaccines for immune activation, and precision diagnostic and therapeutic technologies represents complementary advances in PDAC. While each approach has demonstrated independent clinical value, their integration remains an important future direction that may provide a comprehensive framework for improving outcomes in PDAC.

## 1 Introduction

Pancreatic ductal adenocarcinoma (PDAC) is one of the most formidable challenges in modern oncology, with approximately 60,430 new diagnoses expected annually in the United States and a rapidly increasing global incidence (1). Despite intensive research efforts, the five-year survival rate has remained below 10% for decades, and PDAC is projected to become the second most common cause of cancer mortality by 2030 (2). This poor prognosis reflects unique biological characteristics that distinguish PDAC from other solid malignancies and profound limitations of current therapeutic approaches.

The challenges inherent in PDAC treatment stem from multiple interconnected factors creating a highly challenging therapeutic environment. Most patients present with advanced, unresectable disease due to early metastatic spread and absence of reliable screening methods (3). The biological complexity of PDAC creates formidable therapeutic barriers through its dense stromal architecture, immunosuppressive environment, and nearly universal KRAS mutations that collectively generate a treatment-resistant phenotype (4, 5). Despite significant technological advances, therapeutic progress has been constrained by fragmented approaches that address individual biological barriers in isolation and fail to account for the considerable adaptability and heterogeneity of this malignancy (6).

Recent technological advances have generated multiple complementary approaches for transforming PDAC management. The development of organoid culture systems enable high-throughput drug screening (7, 8). Patient-derived organoids have demonstrated strong predictive value for clinical outcomes, with drug sensitivity profiles correlating closely with patient treatment responses (9, 10). In parallel, personalized mRNA neoantigen vaccines show meaningful clinical benefits in early-phase clinical studies (11, 12). Advanced technologies including single cell sequencing, spatial transcriptomics, and proteomics provide insights into tumor heterogeneity and resistance mechanisms (13, 14). This review examines these complementary advances in PDAC, whose long-term progression may provide a novel framework that may transform PDAC from an invariably fatal malignancy to a potentially manageable chronic disease.

## 2 Current clinical landscape: standard-of-care and precision therapies

The therapeutic landscape for PDAC has evolved significantly through landmark clinical trials. The FOLFIRINOX regimen demonstrated superior overall survival compared with gemcitabine monotherapy (11.1 vs. 6.8 months), establishing combination chemotherapy as the foundation for treating metastatic PDAC in fit patients (15). Subsequently, nab-paclitaxel plus gemcitabine showed improved overall survival (8.5 vs. 6.7 months), providing an alternative regimen (16). The recent NAPOLI-3 trial demonstrated the superiority of NALIRIFOX over nab-paclitaxel plus gemcitabine, establishing a new benchmark for first-line treatment with median overall survival of 11.1 months versus 9.2 months (17).

FOLFIRINOX has also shown significant benefits in the adjuvant setting. The PRODIGE 24 trial demonstrated that adjuvant FOLFIRINOX improved disease-free survival compared with gemcitabine (21.6 vs. 12.8 months) in patients with resected pancreatic cancer (18). The treatment paradigm for localized PDAC has shifted significantly toward neoadjuvant therapy for borderline resectable and locally advanced disease, with recent randomized trials demonstrating improved resection quality and compliance (19).

Significant strides have been made in precision medicine. For patients with germline BRCA1/2 mutations, the PARP inhibitor olaparib improves progression-free survival (7.4 vs. 3.8 months) (20). Recent FDA approvals have expanded targeted therapy options including KRAS G12C inhibitors such as sotorasib and adagrasib, which have shown response rates of 10-15% in patients with KRAS G12C-mutant PDAC (21). **Table 1** summarizes these pivotal clinical trials that have shaped the current therapeutic landscape. These successes underscore the importance of genomic profiling in guiding treatment and highlight the emerging role of personalized approaches in PDAC management.

**Table 1:** Pivotal clinical trials and therapeutic advances in pancreatic ductal adenocarcinoma.

Treatment category	Agent/regimen	Clinical trial	Patient population	Median OS/key endpoint	Key findings	Year
First-line chemotherapy	FOLFIRINOX	PRODIGE/ACCORD11	Metastatic PDAC	OS: 11.1 vs 6.8 months	Superior to gemcitabine monotherapy; established combination standard	2011
First-line chemotherapy	Nab-paclitaxel + Gemcitabine	MPACT	Metastatic PDAC	OS: 8.5 vs 6.7 months	Alternative to FOLFIRINOX	2013
First-line chemotherapy	NALIRIFOX	NAPOLI-3	Metastatic PDAC	OS: 11.1 vs 9.2 months	New first-line standard for fit patients	2023
Adjuvant therapy	FOLFIRINOX	PRODIGE 24	Resected PDAC	DFS: 21.6 vs 12.8 months	Improved disease-free survival; new adjuvant standard	2018
Targeted therapy	Olaparib	POLO	BRCA-mutation PDAC	PFS: 7.4 vs 3.8 months	First PARP inhibitor maintenance therapy	2019
Targeted therapy	KRAS G12C inhibitors	Phase I/II	KRAS G12C-mutant PDAC	ORR: 10-15%	First KRAS-targeted therapy; limited efficacy	2023
Immunotherapy	Autogene cevumeran + atezolizumab	Phase I	Resected PDAC	RFS: NR vs 13.4 months*	Novel mRNA-based immunotherapy	2023
Immunotherapy	TG01/GM-CSF	Phase I/II	RAS-mutated PDAC	OS: 33.1 months	Peptide-based vaccine with prolonged survival	2020
Immunotherapy	ELI-002 2P (KRAS vaccine)	AMPLIFY-201	KRAS G12D/R-mutant PDAC	mRFS: 16.3 months; mOS: 28.9 months	Off-the-shelf KRAS vaccine; 84$ immunogenicity	2024
Immunotherapy	Pembrolizumab	KEYNOTE-158	MSI-high/dMMR PDAC	ORR: 18.2% in PDAC cohort	FDA-approved for MSI-high tumors	2020
Immunotherapy	Anti-PD-1/PD-L1 agents	AGEO European Cohort	MSI-high/dMMR PDAC	ORR: 48.4%; DCR: 67.7%; mPFS: 26.7 months	High efficacy in MSI-high subset across various ICIs	2023
Immunotherapy	Durvalumab + Tremelimumab	Phase II	Metastatic PDAC (MSS)	ORR: 3.1%; mPFS: 1.5 months	Dual checkpoint inhibition ineffective in MSS PDAC	2019
Immunotherapy	CY/GVAX + Nivolumab + SBRT	Phase II	Borderline resectable PDAC	mOS: 20.4 months	Neoadjuvant vaccine plus checkpoint inhibitor combination	2023
Immunotherapy	CY/GVAX + Pembrolizumab + SBRT	Phase II	Locally advanced PDAC	resection rate: 44%	Neoadjuvant immunotherapy with improved resectability	2021
Immunotherapy	Dendritic cell vaccines (GVAX-DC)	Phase I/II	Resected PDAC	mOS: 24.8 months	DC-based immunotherapy with adjuvant potential	2024

*Recurrence-free survival of responders versus non-responders. OS, overall survival; DFS, disease-free survival; PFS, progression-free survival; RFS, recurrence-free survival; ORR, objective response rate; NR, not reached; PDAC, pancreatic ductal adenocarcinoma.

Immunotherapy represents an emerging treatment modality in PDAC despite the traditionally immunosuppressive tumor microenvironment. KRAS targeted peptide vaccines, including TG01/GM-CSF and ELI-002 2P, have demonstrated encouraging survival and immunogenicity outcomes (22, 23). Neoadjuvant combinations of allogenic tumor vaccines with checkpoint inhibitors and radiotherapy have improved resectability in advanced disease (24, 25). For patients with microsatellite instability-high tumors (1-2% of PDAC), pembrolizumab has achieved FDA approval (26, 27). In contrast, dual checkpoint inhibition proved ineffective in microsatellite-stable (MSS) PDAC, which comprise the majority of cases, underscoring the profound immunosuppressive nature of the PDAC microenvironment (28). Although these approaches remain investigational, they represent important therapeutic developments summarized in **Table 1** and discussed in detail in Section 5.2.

## 3 Biological foundations of PDAC resistance

### 3.1 Intratumor heterogeneity and multi-layered resistance

PDAC exhibits significant cellular diversity within individual tumors, posing major challenges to treatment efficacy (6). Intratumor heterogeneity (ITH) encompasses genetic and metabolic variations that enable tumor evolution and adaptation (29, 30). This heterogeneity is actively maintained through crosstalk between tumor cells and the stromal microenvironment (31), creating distinct cellular subpopulations with varying sensitivities to therapeutic agents that serve as reservoirs for chemoresistance (32).

Therapeutic resistance arises through multiple interconnected mechanisms operating across different temporal and spatial scales (33). Genetic evolution drives the emergence of resistant tumor clones through acquisition of mutations in drug targets and bypass pathways, whereas epigenetic reprogramming enhances cellular plasticity and adaptability (32). Concurrently, microenvironmental adaptations mediated by stromal-immune interactions create physical and biological barriers to therapy (4, 34). Cancer-associated fibroblasts (CAFs) contribute significantly to chemoresistance by secreting exosome-derived mi croRNAs that reprogram tumor cell metabolism. CAF-derived miR-3173-5p specifically targets ACSL4 to suppress ferroptosis and confer gemcitabine resistance (35). PDAC cells employ autophagy-dependent mechanisms to evade immune surveillance by selectively degrading MHC-I molecules, effectively rendering tumor cells invisible to CD8+ T cells (36). These stromal interactions are further reinforced by ERK-dependent transcriptional landscapes that drive PDAC growth and therapeutic resistance (37).

Rather than pursuing traditional maximum tolerated dose approaches that may select for resistant clones, recent evidence suggests that constraining cellular heterogeneity through combination approaches represents a more viable strategy for halting PDAC progression (6). The goal shifts from eliminating all tumor cells to constraining their evolutionary potential and rendering them vulnerable to conventional therapies (38).

### 3.2 Immunosuppressive microenvironment

The PDAC microenvironment is characterized by multiple immunosuppressive cell populations that collectively create a hostile environment for antitumor immunity (4). Myeloid-derived suppressor cells (MDSCs) constitute a key element, facilitating PDAC progression and inhibiting effective antitumor immunity (39). These cells mediate resistance to treatment through T cell suppression and enhanced vascular formation (40, 41). The recruitment and activation of MDSCs involves complex molecular mechanisms, including the CRIP1/NF-κB/CXCL axis, where CRIP1 facilitates NF-κB nuclear translocation leading to CXCL1/5 secretion that drives MDSC chemotaxis and establishes immunosuppressive niches (42). PDAC-derived factors promote metabolic reprogramming of MDSCs, enhancing their immunosuppressive capacity (43, 44).

Single-cell transcriptomic analysis has identified distinct CAF subtypes that differentially contribute to PDAC pathogenesis, including inflammatory CAFs (iCAFs) characterized by high expression of inflammatory mediators and myofibroblastic CAFs (myCAFs) distinguished by elevated alpha-smooth muscle actin expression (45, 46). Recent studies have uncovered an interferon-response CAF (ifCAF) subtype with tumor-restraining properties that can be induced by STING agonists and can suppress tumor cell invasiveness (47, 48). Epigenetic alterations also play crucial roles, with loss of SETD2 leading to ectopic H3K27 acetylation that drives BMP2 signaling and promotes lipid-laden CAF differentiation (49). Stromal signals orchestrate epigenetic reprogramming through histone modifications, creating a BRD2-dependent regulatory hub that integrates oncogenic KRAS pathways with microenvironmental cues (50).

## 4 Diagnostic and predictive technologies

### 4.1 Molecular subtyping and spatial technologies

Molecular subtyping has identified distinct transcriptional programs that correlate with clinical outcomes and treatment responses. The classical and quasi-mesenchymal subtypes show differential responses to therapy, with classical tumors generally demonstrating better survival outcomes (51). Subsequent work revealed that pancreatic tumors contain independent tumor- and stroma-derived molecular signatures, with the worst clinical outcomes observed when basal-like tumor features co-occur with an activated stromal microenvironment (52). Recent consensus molecular subtyping approaches have integrated both tumor and microenvironmental features to provide a more comprehensive classification system (53). Spatial transcriptomic analysis reveals complex tissue architecture and distinct cellular neighborhoods within tumors (54). Integration with single-cell RNA sequencing uncovers cellular subtypes involved in aggressive features, though translating findings into therapeutic strategies remains challenging (14, 55).

### 4.2 Organoid models and liquid biopsy

Patient-derived tumor organoids (PDTOs) are three-dimensional culture systems that maintain key features of primary tumors, including genetic, histological, and drug response characteristics (10, 56). Drug sensitivity profiles of PDOs closely mirror patient treatment responses, demonstrating strong clinical predictive value (9, 57). Integration with advanced tools, such as label-free single-cell phenotyping, enables real-time assessment of tumor heterogeneity (58). These systems have identified novel therapeutic targets, exemplified by the discovery of perhexiline maleate, an inhibitor of mutant KRAS through cholesterol synthesis modulation (59).

PDAC organoids enable high-throughput screening and systematic evaluation of therapeutic compounds, with recent advances focusing on developing standardized protocols for organoid culture, drug screening assays, and data interpretation (60, 61). The integration of organoid screening with genomic profiling enhances the precision of treatment selection, enabling clinicians to predict chemotherapy resistance and improve therapeutic efficacy (8, 10).

Patient-derived organoids preserve transcriptional heterogeneity observed in primary tumors, with studies demonstrating that PDOs can maintain the coexistence of ‘classical’ and ‘basal-like’ transcriptional subtypes within individual organoid cultures (62). Single-cell transcriptomic analyses indicate this preserved cell state plasticity may influence therapeutic responses, potentially enabling investigation of subtype-dependent drug sensitivity patterns (63). From a practical perspective, PDOs can be established from endoscopic ultrasound-guided fine-needle biopsy (UES-FNB) specimens with 70-87% success rate, providing a less invasive approach for unresectable cases (64). Cell-free DNA isolated from organoid culture supernatants as early as 72 hours post-biopsy can reflect the mutational profile of primary tumors, suggesting potential for rapid molecular characterization (65). Additionally, emerging data suggest that PDO morphology may correlate with molecular subtypes and should be classified within 2 weeks of tissue sampling (66). While these morphological patterns have shown association with treatment response and overall survival in initial studies, the predictive value of morphological subtyping requires validation in larger cohorts before routine clinical implementation can be recommended.

While PDOs offer significant advantages for personalized medicine, important limitations must be acknowledged that currently constrain their clinical implementation. The progressive loss of stromal components during serial passaging depletes immune cells, cancer-associated fibroblasts, and endothelial (67–69) This loss of tumor microenvironment interaction may result in incomplete recapitulation of drug responses that are modulated by stromal or immune factors, potentially limiting the ability to evaluate immunotherapies or agents targeting the tumor-stroma interface. Standard PDO cultures require sophisticated co-culture systems with patient-matched immune cells to adequately assess the immunotherapy responses (70). To address these limitations, ongoing research efforts are focused on developing complex organoid models that integrate immune components, vascular structures, and stromal elements to better recapitulate the native tumor microenvironment. Turnaround time of 3–4 weeks from tissue acquisition to drug screening results (71, 72) may exceed decision making windows for rapidly progressing disease. Technical establishment rates vary considerably across protocols and tissue sources, ranging from 20% to 90% depending on sample quality, biopsy technique, and culture methodology (73). This variability introduces potential selection bias, as failure may preferentially occur with less proliferative or more differentiated tumor subtypes.

Liquid biopsy has emerged as a valuable approach in oncology, offering noninvasive molecular profiling capabilities. Recent developments have focused on integrating multi-analyte liquid biopsy panels to provide comprehensive tumor profiling (74), enabling precision medicine by allowing clinicians to track molecular changes in real-time (75). Liquid biopsy technologies have demonstrated particular promise in pancreatic cancer due to inherent challenges of tissue acquisition (76). Circulating tumor DNA (ctDNA) analysis enables noninvasive and dynamic monitoring of tumor burden and treatment response in pancreatic cancer patients (77). Surveillance programs utilizing advanced diagnostic technologies have demonstrated significant improvements in outcomes, with surveillance-detected PDAC showing better survival rates and earlier-stage diagnoses (78). While promising, significant challenges remain in standardizing protocols, improving sensitivity for early-stage disease, and demonstrating clinical utility through prospective trials.

## 5 Therapeutic innovations

### 5.1 KRAS-targeted and metabolic therapies

Large-scale genomic investigations demonstrated the prognostic importance of distinct KRAS mutations in patients with PDAC. In a significant analysis of 1,360 patients who underwent surgical treatment, tumors harboring KRAS G12R mutations were more frequently observed in early-stage cases and demonstrated reduced rates of distant metastasis along with superior overall survival outcomes relative to other KRAS mutation subtypes (79). The development of KRAS-targeted therapies has shown promising clinical results, with several inhibitors demonstrating anticancer activity in clinical trials (80). These developments represent significant advances in PDAC treatment, overcoming the long-standing challenge of targeting KRAS, which has emerged as a tractable therapeutic target (81). However, clinical experience remains limited, with most data from early-phase trials, and questions persist regarding optimal patient selection, resistance mechanisms, and long term efficacy.

PDAC cells undergo profound metabolic reprogramming to survive within a uniquely hypoxic and nutrient-scarce tumor microenvironment, creating targetable vulnerabilities. PDAC cells exhibit distinct metabolic subtypes characterized by enhanced glycolysis, glutaminolysis, and altered lipid metabolism, which correlate with differential sensitivities to specific metabolic inhibitors (82). Although autophagy suppression through chloroquine or hydroxychloroquine showed therapeutic potential in laboratory studies, randomized clinical trials have revealed limited clinical benefits attributed to adaptive upregulation of alternative nutrient scavenging mechanisms (83). Recognizing this compensatory response, researchers have developed combination strategies that simultaneously target both autophagic recycling and macropinocytic uptake pathways, resulting in enhanced antitumor efficacy in experimental PDAC models (84, 85). Additionally, disrupting glutamine metabolism using glutaminase inhibitors has shown promise in KRAS-driven PDAC models, particularly when combined with modulation of the NRF2-KEAP1 signaling axis (86, 87).

### 5.2 mRNA vaccines and immunotherapy

The development of personalized mRNA neoantigen vaccines represents a notable advance in PDAC immunotherapy. These vaccines deliver tumor-specific antigenic sequences to antigen-presenting cells, which then prime naïve T cells to recognize and eliminate cancer cells bearing these neoantigens, inducing potent neoantigen-specific cytotoxic T cell responses (88). This mechanism leverages lipid nanoparticle-encapsulated mRNA, which is internally translated into target proteins, processed into peptides, and presented on MHC molecules to activate T cell responses. Early-phase clinical data for the adjuvant autogene cevumeran have shown significant clinical benefits, with vaccine-induced immune responses serving as a strong predictor of improved disease-free survival outcomes (11). Patients with vaccine-expanded T cells showed significantly prolonged recurrence-free survival compared to non-responders (median not reached vs. 13.4 months, P = 0.003).

Contemporary vaccine development has expanded beyond mRNA platforms to include diverse therapeutic modalities. The AMPLIFY-201 trial offered initial clinical evidence supporting the potential of standardized peptide vaccines targeting recurrent KRAS mutations and highlighted their ability to induce mutation-specific immune responses with acceptable safety (22). The TG01/GM-CSF vaccine targeting KRAS oncogenic mutations demonstrated high immunogenicity when combined with adjuvant gemcitabine in patients with resected pancreatic adenocarcinoma, with over 90% of patients showing positive immune responses and a median overall survival of 33.1 months (23). These findings support the continued development of multiple vaccine platforms with personalized mRNA vaccines leading the way toward precision immunotherapy of PDAC.

## 6 Discussion

PDAC represents a formidable oncological challenge with profound therapeutic resistance and poor outcomes. Recent biotechnological and clinical advances have created multiple complementary approaches toward improvement. The development of PDTOs that can accurately predict individual therapeutic responses (10, 56), clinical validation of personalized mRNA neoantigen vaccines capable of inducing robust antitumor immunity (11), and successful targeting of the KRAS oncogene (21, 89) collectively represents significant progress in PDAC research and treatment. Advanced diagnostic technologies such as spatial transcriptomics and multi-analyte liquid biopsies may offer a high-resolution perspective of tumor biology (52, 54, 76). Nevertheless, several critical barriers must be overcome before these technologies can be routinely integrated into clinical practice, including standardization of methods, and validation across diverse patient populations.

The development of patient-derived organoids and personalized mRNA neoantigen vaccines exemplifies complementary approaches in PDAC precision medicine. Organoids enable prospective identification of effective therapies through ex vivo drug screening, demonstrating strong concordance with clinical responses (10, 61), while mRNA vaccines extend precision medicine principles to immune-based relapse prevention by generating tumor-specific T cell responses, with vaccine responders showing significantly prolonged recurrence-free survival (11). These platforms address different stages of the therapeutic continuum—organoids for treatment selection and vaccines for adjuvant immunotherapy—yet both rely on patient-specific molecular information. Proof-of-concept studies have demonstrated that organoid-derived genomic profiles comparable to primary tumors (90, 91), which could theoretically inform rational neoantigen selection for vaccine design. However, clinical integration of these complementary platforms remains to be systematically validated. Future investigations are warranted to determine whether such integrated approaches can improve therapeutic outcomes beyond the independent application of each technology (92).

The clinical feasibility and therapeutic relevance of these approaches are no longer theoretical. Evidence from clinical trials indicates that patients harboring actionable molecular alterations have substantially improved median overall survival when treated with matched therapies, reinforcing the clinical validity of precision oncology approaches (93). Prospective studies such as COMPASS have shown that comprehensive genomic profiling can be delivered within a clinically actionable timeframe, with treatment outcomes strongly influenced by genomic and transcriptomic subtypes (94). Current ASCO guidelines emphasize routine molecular testing for microsatellite instability, BRCA mutations, and other actionable alterations to guide targeted therapies and immunotherapy (95).

While these technologies are advancing independently, their coordinated integration could form the basis for future strategies aimed at more comprehensive therapeutic management. The historical approach of applying static, one-size-fits-all regimens for biologically heterogeneous diseases is becoming obsolete (92). Emerging AI-driven platforms provide novel analytical capabilities for integrating complex datasets. AI-assisted analysis of temporal imaging from PDTO co-culture systems enables dynamic assessment of therapeutic responses (96), while machine learning algorithms integrate multi-omics data to enhance treatment prediction (97). These AI-based prognostic models demonstrate superior predictive performance compared to conventional staging approaches (98).

Nevertheless, significant challenges must be addressed. Analysis of refractory metastatic cancers has revealed that standard-of-care resistance biomarkers are identified in only 9.6% of treatment-resistant tumors, highlighting the urgent need for broader validation of investigational resistance mechanisms (99). As we deploy these novel therapies, we must anticipate and study the next generation of resistance mechanisms, such as acquired mutations that bypass KRAS inhibition (100) or immunoediting that allows tumors to escape vaccine-induced T cell responses (101, 102). The PDAC biology requires multifaceted therapeutic approaches including radiation-enhanced immunotherapy (103), dendritic cell-based immunotherapeutic strategies (24), mRNA vaccines with conventional chemotherapy (101), and organoid-guided personalized therapy with adaptive dosing strategies (71). Manufacturing scalability represents a critical bottleneck in personalized therapies, with mRNA vaccine production timelines requiring further optimization for routine clinical implementation (104).

In conclusion, the parallel development of patient-derived organoids for drug sensitivity testing, personalized mRNA vaccines for immune activation, and precision diagnostic technologies provides complementary tools that address different aspects of DPAC biology. While each approach has demonstrated independent clinical value, their long-term integration may provide a conceptual basis for more coordinated strategies in PDAC management. Although the prospect of converting pancreatic cancer from an invariably fatal diagnosis into a manageable chronic condition remains highly challenging, continued technological and therapeutic advances suggest incremental progress toward this goal. Achieving this vision will require sustained collaborative efforts from basic scientists, clinical researchers, bioinformaticians, regulatory bodies, and pharmaceutical partners to translate these significant scientific advances into meaningful improvements in patient outcomes.
